# Combination of Low Concentration of (−)-Epigallocatechin Gallate (EGCG) and Curcumin Strongly Suppresses the Growth of Non-Small Cell Lung Cancer in Vitro and in Vivo through Causing Cell Cycle Arrest

**DOI:** 10.3390/ijms140612023

**Published:** 2013-06-05

**Authors:** Dong-Hu Zhou, Xuemin Wang, Mingmin Yang, Xiaoyan Shi, Wenbin Huang, Qing Feng

**Affiliations:** 1 Department of Nutrition and Food Safety, Nanjing Medical University, Nanjing 211166, Jiangsu, China; E-Mails: donghuzhou@gmail.com (D.-H.Z.); wangxuemin0303@163.com (X.W.); yangmingmin728@163.com (M.Y.); xiaoyanshi321@163.com (X.S.); 2 Department of Pathology, Affiliated Nanjing First Hospital of Nanjing Medical University, Nanjing 210006, Jiangsu, China; E-Mail: wbhuang348912@126.com

**Keywords:** EGCG, curcumin, cell cycle arrest, combination, chemoprevention

## Abstract

(−)-Epigallocatechin gallate (EGCG) and curcumin are two naturally derived agents that have been widely investigated worldwide. They exhibit their anti-tumor effects in many types of cancers. In the current study, the effect of the combination of the two agents on non-small cell lung cancer (NSCLC) cells was investigated. The results revealed that at low concentrations, the combination of the EGCG and curcumin strongly enhanced cell cycle arrest. Flow cytometry analysis showed that the cells were arrested at G1 and S/G2 phases. Two main cell cycle related proteins cyclin D1 and cyclin B1 were significantly inhibited at the present of EGCG and curcumin. EdU (5-ethynyl-2′-deoxyuridine) fluorescence staining showed that the DNA replication was significantly blocked. A clonal growth assay also confirmed a marked repression of cell growth. In a lung cancer xenograft node mice model, combination of EGCG and curcumin exhibited protective effect against weight loss due to tumor burden. Tumor growth was strongly repressed by the combination of the two agents, without causing any serious side-effect. Overall, these results strongly suggest that EGCG in combination with curcumin could be a candidate for chemoprevention agent of NSCLC.

## 1. Introduction

**Figure 1:**
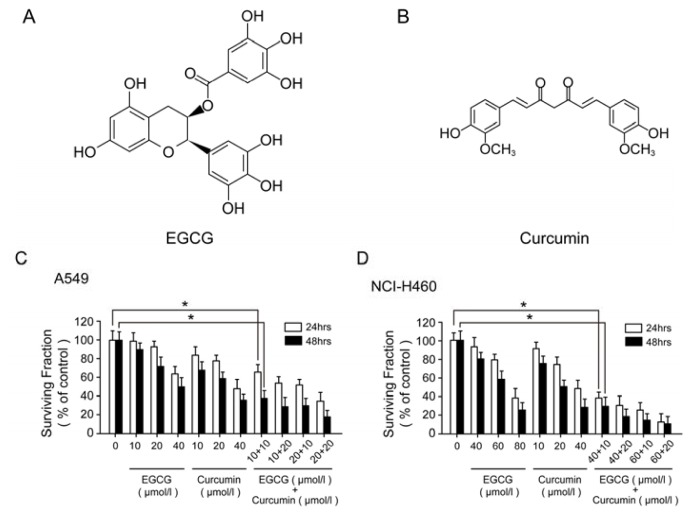
Combination of (−)-Epigallocatechin gallate (EGCG) with curcumin caused significant growth inhibition in A549 and NCI-H460 cells. (**A**,**B**) The chemical structure of EGCG and curcumin respectively; (**C**) MTT assay to measure survival fraction in A549 cells after the indicated treatment; (**D**) MTT assay to measure survival fraction in NCI-H460 cells after the indicated treatment (* *p* < 0.05).

Lung cancer is a leading cause of cancer death worldwide. Despite improvements in chemotherapy, radiation therapy and surgery, the prognosis of non-small cell lung cancer (NSCLC) remains poor [1]. Moreover, recurrence of this disease is very frequent, suggesting that it is important to keep on looking for safe and effective treatments. In addition to smoking cessation, chemoprevention is an attractive approach. Chemoprevention can be achieved by using natural or synthetic compounds to prevent the occurrence of cancer, to treat early stage cancer and to block the metastatic spread. Dietary chemicals are important chemopreventive agents. It is estimated that nearly one-third of cancer deaths could be prevented through appropriate dietary modification [2].

(−)-Epigallocatechin-3-gallate (EGCG, Figure 1A) is the major catechin found in green tea and has been intensively studied as a chemopreventive agent [3,4]. It has shown anti-cancer effect (including prostate, head and neck, and lung cancer) in many *in vitro* studies and animal models [5–7]. Generally, EGCG can block a series of signal transduction pathways related to carcinogenesis [8,9], and acts as an inhibitor of receptor tyrosine kinase and proteasome [3,4]. Also, EGCG can inhibit or down-regulate DNA methyltransferases (DNMTs) [7]. Another well-studied chemopreventive compound is curcumin (Figure 1B), which is the major yellow pigment in turmeric. Curcumin also shows its anti-tumor effects in multiple cancer cell lines and animal models [10–12]. It is reported that curcumin induces the inhibition of several cell signaling pathways at multiple levels, such as transcription factors, enzymes, cell cycle arrest, proliferation, survival pathways and TNF [13]. Curcumin can up-regulate caspase family proteins and down-regulate anti-apoptotic genes [13]. By using cDNA microarrays, studies have demonstrated curcumin can act at the genomic anti-tumor level in leukemia and lung cancer [14,15].

Cell cycle as a therapeutic target is gaining more and more attention [16]. The cell cycle offers a multitude of prognostic, predictive and therapeutic possibilities, though many of which are still in the developing stage [17]. Most NSCLCs have detectable cell cycle abnormalities. Many recent studies demonstrated that EGCG could trigger cell cycle arrest at the G1 phase through regulation of cyclin D1, cdk4, cdk6, p21/WAF1/CIP1 and p27/KIP1 [18]. In multiple cancer cell lines, EGCG blocks cell cycles at the G0/G1 phase, and then suppresses cell proliferation and invasion [9,19]. In comparison with EGCG, curcumin inhibits cell proliferation and cell cycle progression by accumulating cells in S and G2/M phases [20,21]. Actually, the anti-tumor effect of curcumin has been attributed in part to the arrest of malignant cells in S, G2/M phases and subsequently induction of apoptosis [22].

The approach of combination therapy has been used successfully in the treatment of several types of cancer [23]. It is effective to achieve higher therapeutic efficacy with lower drug dosage and reduce drug resistance development [24]. Similarly, combinations of naturally derived agents may produce better chemopreventive effects. In breast cancer and malignant human oral epithelial cells, when EGCG and curcumin were given in combination, they synergistically induced apoptosis *in vitro* and *in vivo* [25,26].

In the current study, we investigated whether combination of EGCG and curcumin would produce higher inhibitory activity against NSCLC cells *in vitro* and *in vivo*. We analyzed the synergistic effect of EGCG combined with curcumin on cell proliferation, cell cycle arrest and DNA replication in NSCLC cell lines. We found that, using a much lower concentration than reported before, the inhibition of cell growth was mostly caused by the enhanced cell cycle arrest, instead of inducing apoptosis. The same conclusion was also drawn in a xenograft model in nude mice. Considering the poor absorption and low bioavailability of curcumin, its combination with EGCG may increase the efficacy for lung cancer prevention.

## 2. Results

### 2.1. Anti-Proliferation Effect of EGCG and Curcumin in NSCLC Cells

To investigate the effect of EGCG and curcumin on NSCLC cells growth, a dose-course and a time-course experiment were performed in A549 and NCI-H460 cells (Figure 1C,D). The cells were treated with EGCG and curcumin alone or in combination. Cell growth was inhibited in a dose- and time-dependent manner, and only at a higher concentration, could single agent cause a significant inhibition of cell proliferation. Based on this result, lower concentrations of EGCG and curcumin were selected and applied to investigate the synergistic effect of the two agents. Figure 1C,D showed that even at a lower concentration, the combination of the two compounds exhibited a synergistic anti-proliferation effect with a CI value of 0.57713 and 0.67703 respectively in A549 and NCI-H460 cells (Table S1–S4).

### 2.2. EGCG and Curcumin Repressed DNA Replication

**Figure 2:**
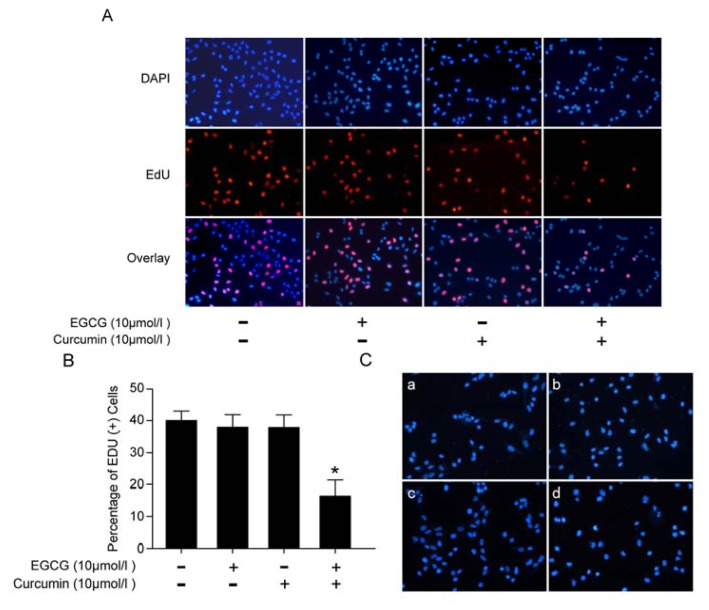
EGCG and curcumin repressed DNA replication in A549 cells. (**A**) EdU fluorescence staining to detect the newly synthesized DNA. The cells were given indicated treatment for 24 h; (**B**) The percentage of EdU (+) cells was calculated by Image-Pro Plus software; (**C**) Hoechst 33258 staining was performed to detect the apoptosis caused by the indicated treatment. (**a**) control; (**b**) treated with 10 μmol/L of EGCG for 24 h; (**c**) treated with 10 μmol/L of curcumin for 24 h; (**d**) treated with 10 μmol/L of EGCG and 10 μmol/L of curcumin for 24 h. (* *p* < 0.05).

The EdU (5-ethynyl-2′-deoxyuridine) fluorescence staining is an indicator of DNA synthesis, which was used to detect newly synthesized DNA in A549 cells after the indicated treatment. DAPI (4′,6-diamidino-2-phenylindole) was used to mark all the nuclei. Results showed that the EdU positive cells in the combination treatment group were much less than in the control group, which meant that the DNA replication was blocked significantly (Figure 2A,B).

Since cell cycle arrest and apoptosis are closely related to cell growth, we next investigated whether the combination of the two agents caused apoptosis in A549 cells. It was reported that, at high concentrations, EGCG and curcumin caused remarkable apoptosis in the breast cancer cells, especially when administrated together [26]. Here, in the experimental condition, which used much lower concentrations, the Hoechst 33258 staining suggested that, after the 24 h treatment, the two agents failed to cause significant apoptosis in A549 cells (Figure 2C).

### 2.3. Combination of EGCG and Curcumin Caused Significant Cell Cycle Arrest

**Figure 3:**
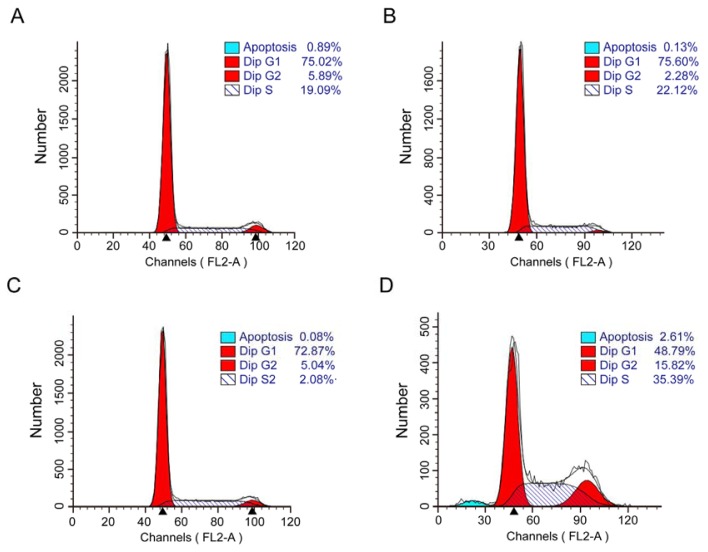
The combination of EGCG and curcumin caused significant cell cycle arrest. Flow cytometric analysis to detect cell cycle. After A549 cells were given indicated treatment for 24 h, cells were harvested and the distribution of cell cycle was exhibited by flow cytometric analysis. (**A**) Control; (**B**) Treated with 10 μmol/L of EGCG; (**C**) Treated with 10 μmol/L of curcumin; (**D**) Treated with 10 μmol/L of EGCG and 10 μmol/L of curcumin.

**Figure 4:**
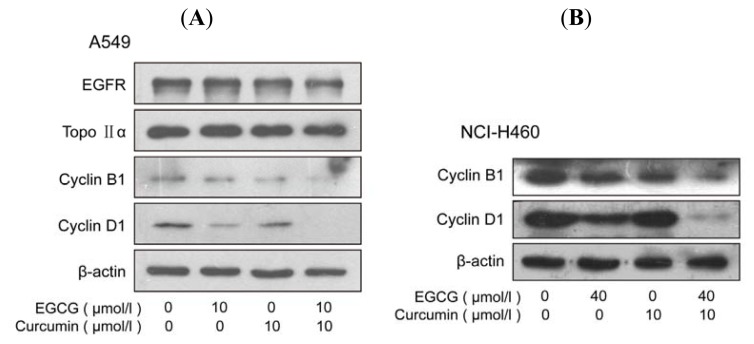
EGCG and curcumin significantly inhibited the expression of cell cycle related protein cyclin D1 and cyclin B1 in NSCLC cells. (**A**) Effect of EGCG and curcumin on cell cycle related proteins in A549 cells; (**B**) Effect of EGCG and curcumin on cell cycle related proteins in NCI-H460 cells. The cells were given indicated treatment for 24 h. Several proteins were measured by Western blotting as described in Materials and Methods. (**A**) (**B**)

Flow cytometry analysis was performed to show the distribution of cell cycle. As it was shown in Figure 3 and Table S5, EGCG in combination with curcumin caused significant cell cycle arrest in A549 cells. The cells arrested in S/G2 phase were highly increased. However, apoptosis only occurred in few cells, consistent with the results in Hoechst 33258 staining. The expression of two main cell cycle related proteins, cyclin B1 and cyclin D1, were investigated by Western blot. Cyclin B1 and cyclin D1 are usually over-expressed in lung cancer cells [16]. Western bolt analysis showed that the expression of cyclin B1 and cyclin D1 (Figure 4A) were strongly repressed at the present of EGCG and curcumin. In NCI-H460 cells, the two agents also had a synergistic effect on the inhibition of expression of cyclin B1 and cyclin D1 (Figure 4B). However, the two agents only had a slight impact on EGFR (Epidermal Growth Factor Receptor) and Topo IIα (Topoisomerase IIα) (Figure 4A), which were connected with cell proliferation.

### 2.4. EGCG in Combination with Curcumin Repressed Clonal Formation of A549 and NCI-H460 Cells

**Figure 5:**
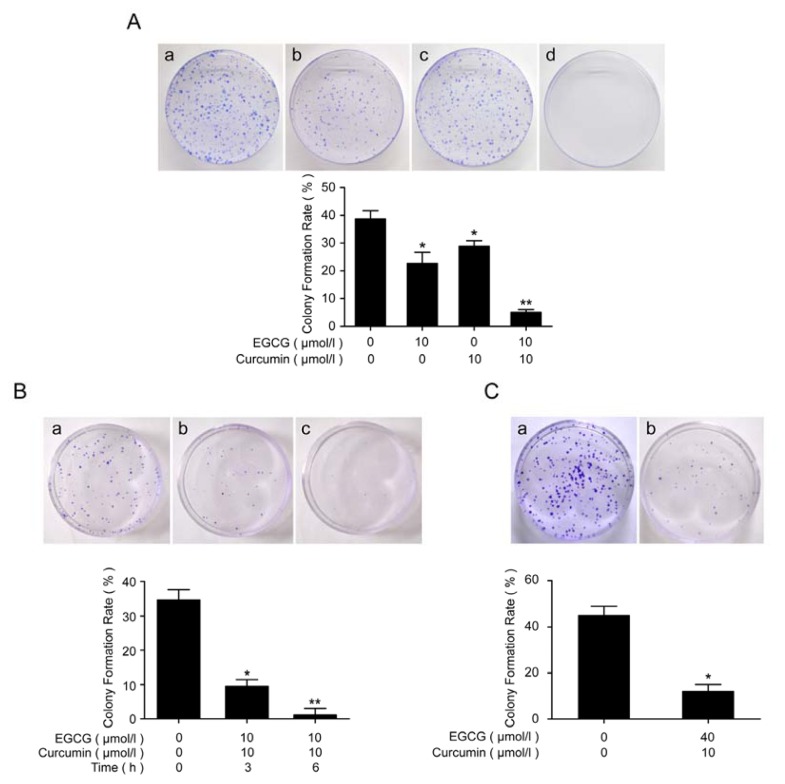
EGCG in combination with curcumin repressed clonal formation of NSCLC cells. (**A**) EGCG in combination with curcumin repressed clonal formation of A549 cells. About 1 × 10^3^ A549 cells were seeded onto the 100 mm plates after the indicated treatment and changed for fresh medium every 3 days. (**a**) control; (**b**) treated with 10 μmol/L of EGCG for 24 h; (**c**) treated with 10 μmol/L of curcumin for 24 h; (**d**) treated with 10 μmol/L of EGCG and 10 μmol/L of curcumin for 24 h; (**B**) EGCG in combination with curcumin repressed clonal formation of A549 cells in a time-dependent manner. About 5 × 10^2^ A549 cells were seeded onto the 60 mm plates after the indicated treatment and changed for fresh medium every 3 days. (**a**) control; (**b**) treated with 10 μmol/L of EGCG and 10 μmol/L of curcumin for 3 h; (**c**) treated with 10 μmol/L of EGCG and 10 μmol/L of curcumin for 6 h; (**C**) EGCG in combination with curcumin repressed clonal formation of NCI-H460 cells. About 1 × 10^3^ NCI-H460 cells were seeded onto the 100 mm plates after the indicated treatment and changed for fresh medium every 3 days; (**a**) control; (**b**) treated with 10 μmol/L of EGCG and 10 μmol/L of curcumin for 24 h. After 7 or 10 days, visible colonies were fixed and stained with crystal violet staining solution. The colony formation rate was calculated (* *p* < 0.05, ** *p* < 0.01).

Effects of EGCG and curcumin on A549 and NCI-H460 cells growth were further studied. In A549 cells, a clonal growth assay confirmed that EGCG in combination with curcumin, conferred a marked repression of clonal growth (Figure 5A). Similar results were obtained in the time-dependent experiment (Figure 5B). In NCI-H460 cells, EGCG and curcumin also strongly inhibited the clonal formation (Figure 5C).

### 2.5. EGCG and Curcumin Inhibited the Tumor Growth in Vivo

**Figure 6:**
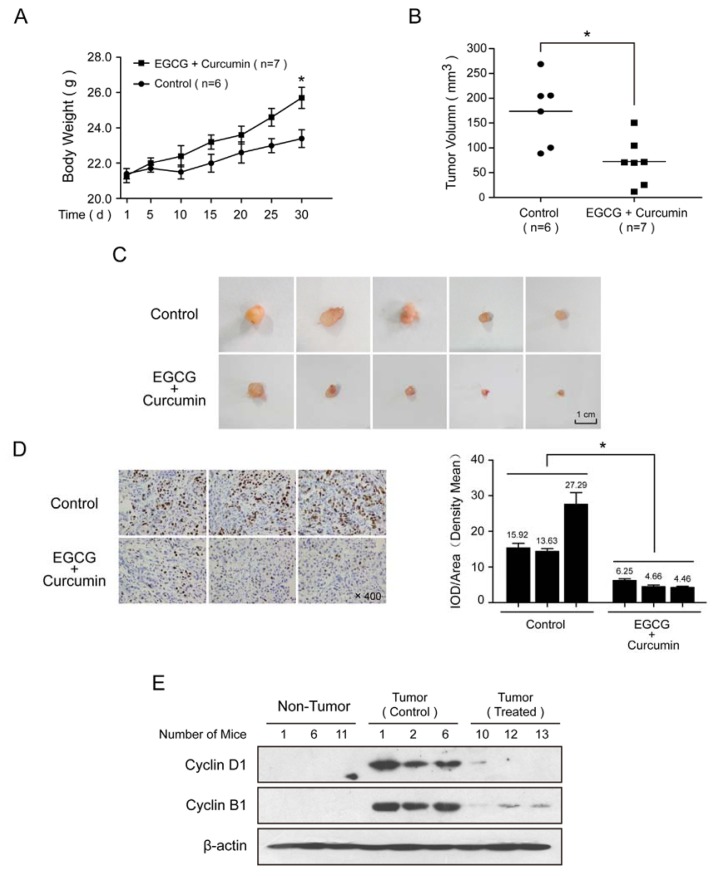
EGCG and curcumin inhibited the tumor growth in lung cancer xenograft nude mice. Fourteen 3 to 4-week old female BALB/c nude mice were i.p. implanted with 5 × 10^6^ A549 cells. At the third day after the A549 cells injected, the mice were randomized into two groups (7 mice/group) and treated with control (NS, 100 mL/kg) or EGCG and curcumin (20 mg/kg, respectively). All the compound(s) was/were administered every other day for 4 weeks through intraperitoneal injection. On the 30th day, all animals were sacrificed. The body weight (**A**) and tumor size (**B** and **C**) were recorded every day; (**D**) **Left**: Immunohistochemistry staining of Ki-67. **Right**: The density mean of Immunohistochemistry staining; (**E**) Western blot was performed to detect the expression of cyclin B1 and cyclin D1. (* *p* < 0.05).

To further investigate whether the low-concentration combination therapy had any impact on tumor progression *in vivo*, NSCLC xenograft nude mice model was developed. A549 cells were injected into the dorsal of the BALB/c nude mice. Since the malignant cells implantation, the body weight of the mice and the tumor size were recorded every day. As was shown in Figure 6A, EGCG and curcumin exhibited a protective effect against the weight loss induced by tumor burden, and the tumor formation in the mice treated by EGCG and curcumin was delayed significantly than the control group (data not shown). After the 30-day treatment, the tumor growth was strongly repressed by EGCG in combination with curcumin, without causing any serious side-effect to the mice (Figure 6B,C). Immunohistochemistry showed that Ki-67 was strongly inhibited in the treated group, which meant that EGCG and curcumin suppressed the tumor growth (Figure 6D). The expression of cyclin B1 and cyclin D1 were further detected between the treated and control group. It appeared that the expression of the two proteins was significantly repressed by EGCG and curcumin (Figure 6E), consistent with the above *in vitro* experiments.

## 3. Discussion

Chemoprevention is a potential anti-cancer approach, which can be achieved by using natural compounds to prevent the occurrence of cancer, or even treat cancer. In the current study, combination of low concentration of two chemopreventive polyphenols, EGCG and curcumin, strongly inhibited A549 cells growth *in vitro* and *in vivo*. To our knowledge, this is the first report to analyze the synergistic suppressive effect EGCG combined with curcumin on NSCLC cells.

In a chemoprevention study, the ideal result would be using the lower concentration of an anti-cancer compound to achieve the best cancer preventive effect. The doses of EGCG and curcumin we focus in this study are extremely low, compared with other similar researches, especially the *in vivo* experiment. Phytochemical such as curcumin, has versatile chemopreventive properties while has poor absorption and low bioavailability [27]. This may restrict its clinical application. It was reported that (−)-epicatechin (EC), another kind of green tea polyphenol, significantly increased the amounts of intracellular curcumin by approximately 1.3-fold more than curcumin itself [26], though the mechanism is still not fully understood yet. Recent studies found that numerous anticancer compounds that presented in edible plants would strengthen their cancer preventive activity when in combination with green tea catechins [28]. Considering the poor absorption and low bioavailability of curcumin, we speculate that EGCG may also enhance the intake and bioavailabity of curcumin when the two agents combined together.

Our preliminary results showed that such low concentration of EGCG or curcmin alone failed to inhibit the tumor growth significantly *in vivo*. Only the combination of the two agents could suppress the tumor growth. It is more important to investigate the synergistic effect *in vivo* than *in vitro*. We will explore the synergistic effect *in vivo* in the future. More studies are required to test the absorption of EGCG and curcumin in animal models and human bodies, figuring out if there is any difference of absorption and bioavailability between being administrated alone or in combination, and the exact mechanisms needs to be investigated.

Cell cycle has been considered as an anti-cancer target for years. Deregulation of cell cycle leads cells proliferate uncontrollably and leads to genetic instability and tumorigenesis, thus can potentially be targeted to inhibit cancer progression [29]. Either EGCG or curcumin is a powerful inhibitor of cell cycle related proteins and cell cycle kinases [18,22]. Combination of the two compounds may simultaneously target multiple cell cycle related pathways. As shown in this study, cyclin B1 and cyclin D1 were inhibited by EGCG combining with curcumin, resulting a significant G1 and S/G2 phases arrest. Meanwhile, the DNA replication was strongly inhibited.

In the current study, we considered the synergistic anti-proliferation effect of EGCG combined with curcumin was mainly caused by the enhanced cell cycle arrest, instead of apoptosis. Since EGCG and curcumin are antioxidants at low concentration [5,13], we speculate that the two agents mainly synergistically target to different cell cycle related proteins. However, with increasing concentrations, EGCG and curcumin may cause oxidative stress in cancer cells, and subsequently apoptosis [30,31]. It was reported that significant apoptosis was observed in ER alpha-breast cancer cells by the combination of EGCG and curcumin [26].

Nowadays, several studies attempted to establish the rational bases for combining cell cycle-specific drug with classical chemotherapy drugs to enhance clinical efficacy [29]. The naturally derived, low-toxicity EGCG and curcumin have their advantages in this field. In another experiment, we successfully observed that EGCG in combination of curcumin made the tumor-burden mice more sensitive to cisplatin treatment: the tumor size was diminished obviously at the present of the two agents. We also noticed that the two agents made the efficacy of cisplatin more stable (unpublished data). Thus, our study indicated that EGCG combines with curcumin could be a potential chemoprevention agent for NSCLC and might benefit patients in future clinical trials.

## 4. Experimental Section

### 4.1. Cell Culture and Reagents

Human non-small cell lung cancer cell A549 and NCI-H460 cells were purchased from Cell Centre of Chinese Academy of Medical Sciences (Beijing, China), and were maintained in Dulbecco′s Modified Eagle Medium (DMEM, GIBCO, Gaithersburg, MD, USA) supplemented with 10% heat-inactivated fetal bovine serum (GIBCO, Gaithersburg, MD, USA) and 1% penicillin/streptomycin (Beyotime Institute of Biotechnology, Shanghai, China). Cells were incubated in a humidified atmosphere of 5% CO_2_ at 37 °C. EGCG and curcumin were purchased from Sigma-Aldrich (St. Louis, MO, USA).

### 4.2. MTT Assay

Cell viability was determined by MTT assay. Cells (2 × 10^3^ per well) were seeded in a 96-well plate and allowed to attach for 24 h. Then EGCG and curcumin were added alone or in combination. After 24 h treatment, cells were incubated with 20 μL of MTT solution (5 mg/mL, AMRESCO, Solon, OH, USA) for 4 h at 37 °C. The MTT formazan crystal was then dissolved in 150 μL DMSO (Lingfeng, Shanghai, China), and the absorbance was measured at 490 nm using a microplate reader (TECAN, Männedorf, Switzerland). A combination index (CI) was calculated from pooled data from 3 individual experiments. The combination index (CI) for two agents were calculated by CompuSyn synergism/antagonism analysis software (Version 1.0, ComboSyn, Inc., Paramus, NJ, USA), which based on the following formula:

$$\text{CI}=\frac{{\text{D}}_{1}}{{\text{D}}_{1x}}+\frac{{\text{D}}_{2}}{{\text{D}}_{2x}}$$

where D_1_ and D_2_ were doses of agents 1 and 2 in combination to achieve *x*% inhibition, whereas D_1_*_x_* and D_2_*_x_* represented doses of compounds 1 and 2 to achieve *x*% inhibition when presented alone. CI values of <1, =1 and >1 indicated respectively synergism, additivity and antagonism in combined agent action [32].

### 4.3. Hoechst 33258 Staining

Cells were seeded in a 6-well plate and allowed to attach for 24 h. Then EGCG and curcumin was added alone or in combination. After 24 h treatment, the cells were fixed in 4% paraformaldehyde for 10 min. After being washed twice with PBS, the cells were stained with 0.5 mL of Hoechst 33258 (Beyotime, Shanghai, China) for 5 min and then again washed twice with PBS. The stained nuclei were observed under an inverted fluorescence microscopy (OLYMPUS, Tokyo, Japan).

### 4.4. EdU Fluorescence Staining

The 5-ethynyl-2′-deoxyuridine (EdU) fluorescence staining was used to detect the newly synthesized DNA in A549 cells after the indicated treatment. All steps were followed the manufacturer′s instructions of Cell-Light EdU DNA cell proliferation kit (RiboBio, Guangzhou, China). The percentage of EDU (+) cells were calculated by Image-Pro Plus software (Version 6.0, Media Cybernetics, Bethesda, MD, USA).

### 4.5. Flow Cytometry Analysis

After cells were treated with EGCG and curcumin either alone or in combination for 24 h, they were harvested and washed twice in cold PBS and fixed in 75% ethanol before assessed for cell cycle profiles. For flow cytometry analysis, A549 cells were washed in PBS, centrifuged and the pellet was incubated with RNase A (Amersham Biosciences, Piscataway, NJ, USA) for 15 min and further incubated with propidium iodide (PI, Sigma-Aldrich, St. Louis, MO, USA) for 2 h at 37 °C. The cell cycle distribution was analyzed by the flow cytometer (BD, San Jose, CA, USA).

### 4.6. Western Blot Analysis

After indicated treatment, cells were harvested and the proteins were extracted followed the manufacturer′s instructions (KeyGEN BioTECH, Nanjing, China). Protein concentration was measured by BCA Protein Assay Kit (Beyotime, Shanghai, China). Identical amounts of protein (40 μg) were boiled for 5 min and separated by SDS-PAGE, then transferred onto a PVDF membrane (Millipore Corporation, Billerica, MA, USA). The membrane was then blocked with 5% non-fat dry milk in Tris-Buffered-Saline with Tween (TBST) for 1 h at room temperature, and incubated with appropriate primary antibodies overnight at 4 °C. After washed with TBST, the membrane was then incubated with appropriate secondary antibody for 1 h at room temperature. After extensive washing with TBST, proteins were visualized by the SuperSignal^®^ West Pico (Thermo Scientific, San Jose, CA, USA). Antibodies used include: anti-cyclin B1 (1:1000 dilution, Cell Signaling Technology, Beverly, MA, USA), anti-cyclin D1 (1:1000, Santa Cruz Biotechnology, Inc., Santa Cruz, CA, USA), anti-EGFR (1:1000, Cell Signaling Technology, Beverly, MA, USA), anti-Topo IIα (1:1000, Bioworld, Louis Park, MN, USA), anti-β-actin (1:1000, BOSTER, Wuhan, China), HRP-Conjugated AffiniPure Goat Anti-Mouse IgG (1:2000, ZSGB-BIO, Beijing, China), HRP-Conjugated AffiniPure Goat Anti-Rabbit IgG (1:2000, ZSGB-BIO, Beijing, China).

### 4.7. Clonal Growth Assay

Clonal growth assay were performed using 1 × 10^3^ and 5 × 10^2^ cells for 100 mm and 60 mm dishes respectively. After the indicated treatment, the cells were seeded in the dishes and changed for fresh medium every 3 days. 7 to 10 days later, visible colonies were fixed and stained with crystal violet staining solution (Beyotime, Shanghai, China).

### 4.8. Xenograft Mouse Model

Female BALB/c nude mice with 3–4 weeks old were purchased from SLAC LABORATORY ANIMAL, China. 5 × 10^6^ A549 cells were injected into the dorsal of the mice, the body weight of the mice and the tumor size were recorded every 3 days. Tumor size was measured using calipers, and volumes were calculated using the following formula: volume (mm^3^) = length × width × width/2. At the third day after the A549 cells injected into the mice, 14 mice were randomized into two groups (7 mice/group) for the following treatments: group1, vehicle; group2, EGCG and curcumin (20 mg/kg, respectively). All agents were administered every other day for 4 weeks through intraperitoneal injection. All animal experiments were performed according to procedures approved by The Center for Hygienic Analysis and Detection.

### 4.9. Immunohistochemistry Staining

Immunohistochemistry staining was performed by Department of pathology, Nanjing Medical College Affiliated Nanjing Hospital. The results of immunohistochemistry staining were analyzed by Image-Pro Plus software (Version 6.0, Media Cybernetics, Bethesda, MD, USA).

### 4.10. Statistical Methods

Data were presented as the mean ± standard deviation (SD). To assess the statistical significance of differences, student′s *t* test was performed. The data were considered statistically significant when the *p* value was less than 0.05.

## 5. Conclusions

In the current study, we found that at a low concentration, the suppression of growth of the NSCLC cells mostly lied on the enhanced cell cycle arrest, instead of inducing apoptosis reported before. We also evaluated the possibility that the two natural compounds as a cancer chemoprevention agent in the A549 xenograft nude mouse model. Considering the poor absorption and low bioavailability of curcumin in clinical trials, we especially focused on a much lower concentration. It was suggested that even at this concentration, EGCG and curcumin could slow down the tumor progression significantly without any serious side effects before tumorigenesis. All these results suggest that EGCG in combination with curcumin could be a candidate for chemoprevention of NSCLC. To our knowledge, this is the first report on the enhanced chemopreventive effect caused by EGCG in combination with curcumin in NSCLC cells.

## Supplementary Information
