# Supplementary material for: Combination of Low Concentration of (−)-Epigallocatechin Gallate (EGCG) and Curcumin Strongly Suppresses the Growth of Non-Small Cell Lung Cancer in Vitro and in Vivo through Causing Cell Cycle Arrest
Source: Int J Mol Sci. 2013 Jun 5;14(6):12023–36. doi: 10.3390/ijms140612023 (PMC3709771; doi:10.3390/ijms140612023)
Supplement: Supplementary file 1 [file ijms-14-12023-s001.pdf]

Supplementary Information

Table S1. The inhibition rate of EGCG and curcumin in A549 cells.

EGCG (μmol/L)	Inhibition rate	Curcumin (μmol/L)	Inhibition rate
10	0.012 ± 0.008	10	0.162 ± 0.031
20	0.071 ± 0.022	20	0.221 ± 0.029
30	0.165 ± 0.028	30	0.388 ± 0.035
40	0.363 ± 0.031	40	0.524 ± 0.027

Table S2. The inhibition rate and CI value of EGCG combined with curcumin in A549 cells.

EGCG (μmol/L)	Curcumin (μmol/L)	Inhibition rate of combination	Combination index (CI)
10	10	0.382 ± 0.038	0.57713
10	20	0.483 ± 0.045	0.69498
20	10	0.526 ± 0.048	0.58945
20	20	0.640 ± 0.043	0.60990

Table S3. The inhibition rate of EGCG and curcumin in H460 cells.

EGCG (μmol/L)	Inhibition rate	Curcumin (μmol/L)	Inhibition rate
40	0.072 ± 0.023	10	0.091 ± 0.019
60	0.214 ± 0.034	20	0.263 ± 0.036
80	0.626 ± 0.038	40	0.517 ± 0.025

Table S4. The inhibition rate and CI value of EGCG combined with curcumin in H460 cells.

EGCG (μmol/L)	Curcumin (μmol/L)	Inhibition rate of combination	Combination index (CI)
40	10	0.621 ± 0.042	0.67703
40	20	0.703 ± 0.048	0.75959
60	10	0.752 ± 0.039	0.76162
60	20	0.881 ± 0.052	0.67159

Table S5. The distribution of cell cycle after the indicated treatment.

	Control	EGCG (10 μmol/L)	Curcumin (10 μmol/L)	Combination
G1 Phase	75.02%	75.60%	72.87%	48.79%
S Phase	19.09%	22.12%	22.08%	35.39%
G2 Phase	5.89%	2.28%	5.04%	15.82%
Apoptosis	0.89%	0.13%	0.08%	2.61%
(Mean)	(23.72)	(23.28)	(22.08)	(22.51)

© 2013 by the authors; licensee MDPI, Basel, Switzerland. This article is an open access article distributed under the terms and conditions of the Creative Commons Attribution license (http://creativecommons.org/licenses/by/3.0/).
